# Tropical leg lymphedema caused by podoconiosis is associated with increased colonisation by anaerobic bacteria

**DOI:** 10.1038/s41598-023-40765-7

**Published:** 2023-08-23

**Authors:** Claudio Neidhöfer, Derick Lekealem Nkwetta, Bangsi Rose Fuen, Njodzeka Flora Yenban, Nancielle Mbiatong, Gordon Takop Nchanji, Patricia Korir, Nina Wetzig, Martin Sieber, Ralf Thiele, Marijo Parcina, Ute Klarmann-Schulz, Achim Hoerauf, Samuel Wanji, Manuel Ritter

**Affiliations:** 1 Institute for Medical Microbiology, Immunology and Parasitology (IMMIP), University Hospital Bonn (UKB), Bonn, Germany; 2 Department of Microbiology and Parasitology, University of Buea, Buea, Cameroon; 3 Research Foundation for Tropical Diseases and the Environment (REFOTDE), Buea, Cameroon; 4 Institute for Functional Gene Analytics, Bonn-Rhein-Sieg University of Applied Sciences, Sankt Augustin, Germany; 5 German Center for Infection Research (DZIF), Neglected Tropical Disease, Partner Site, Bonn-Cologne, Bonn, Germany; 6 German-West African Centre for Global Health and Pandemic Prevention (G-WAC), Partner Site, Bonn, Bonn, Germany

**Keywords:** Chronic inflammation, Skin diseases, Inflammation

## Abstract

The non-filarial and non-communicable disease podoconiosis affects around 4 million people and is characterized by severe leg lymphedema accompanied with painful intermittent acute inflammatory episodes, called acute dermatolymphangioadenitis (ADLA) attacks. Risk factors have been associated with the disease but the mechanisms of pathophysiology remain uncertain. Lymphedema can lead to skin lesions, which can serve as entry points for bacteria that may cause ADLA attacks leading to progression of the lymphedema. However, the microbiome of the skin of affected legs from podoconiosis individuals remains unclear. Thus, we analysed the skin microbiome of podoconiosis legs using next generation sequencing. We revealed a positive correlation between increasing lymphedema severity and non-commensal anaerobic bacteria, especially *Anaerococcus provencensis*, as well as a negative correlation with the presence of *Corynebacterium*, a constituent of normal skin flora. Disease symptoms were generally linked to higher microbial diversity and richness, which deviated from the normal composition of the skin. These findings show an association of distinct bacterial taxa with lymphedema stages, highlighting the important role of bacteria for the pathogenesis of podoconiosis and might enable a selection of better treatment regimens to manage ADLA attacks and disease progression.

## Introduction

Podoconiosis causes bilateral lymphedema of the lower limbs and it is estimated that worldwide around 4 million individuals are affected, especially in Africa but also in parts of Latin America and South East Asia^1–3^. Until now, 32 countries reported cases of podoconiosis with the highest prevalence rates in Ethiopia, Cameroon and Uganda^4–8^. Control and elimination are integrated into lymphatic filariasis morbidity management and disability prevention, but the WHO has not officially recognized podoconiosis as a neglected tropical disease. Risk factors like exposure to volcanic red clay soils, which are formed by weathering of rocks predominately in areas characterised by high rainfall and altitude as well as low temperature, heritability and genetic factors, and profession as a farmer were associated with podoconiosis^9–12^. Nevertheless, environmental and socioeconomic factors cannot be used as strict causes of the disease since podoconiosis has been reported also in areas with low altitude, less rainfall, high temperature and in individuals who were not farmers. Diagnosis of podoconiosis and especially discrimination from other infectious (e.g., co-endemic lymphatic filariasis) and non-infectious lymphedema forms depends on a combination of different diagnostic tools including assessment of medical history and chronic diseases accompanied with a detailed physical examination^5,13^. Diagnosis of podoconiosis requires trained health workers in rural areas of low- and middle-income countries and thus, research on podoconiosis-specific biomarkers needs to be performed to facilitate the diagnosis of the disease.

However, contact with volcanic clay in combination with the major risk factors seem to be the cause of the disease but until now specific component(s)/molecule(s) within the soil that drive podoconiosis remain unknown. The bilateral lymphedema caused by podoconiosis can be divided into five stages according to the de novo clinical staging system postulated by Tekola and colleagues^14^. Moreover, podoconiosis is characterised by nodule formation accompanied with extensive sclerosis, loss of elastic fibres, verrucous acanthosis and changes of eccrine structures^14–16^. Consequently, individuals with podoconiosis suffer from skin lesions that serve as entry points for pathogens like bacteria causing inflammatory episodes called acute dermatolymphangioadenitis (ADLA) attacks driving inflammation, progression of the lymphedema and are characterised by malaise, fever, chills and even skin peeling (scaling off of the skin)^17–19^. However, bacterial composition of the legs of podoconiosis individuals has not been analysed and/or compared to the skin microbiome of filarial-driven lymphedema legs which have been shown to harbour distinct patterns of bacterial species^20,21^. Therefore, this study analysed the bacterial composition on the skin of podoconiosis-driven lymphedema legs in the North West Region (NRW) of Cameroon.

## Results

### Characterisation of the study population

As shown in Table 1, 82 participants were enrolled in the study including 8 lymphedema-free individuals who lived in the same communities and 9 individuals with one healthy leg (n = 25 stage 0). The majority of individuals were women (n = 66, 80.49%) and worked as farmers (n = 47, 57.32%). The median age of the participants was 43.2 years and most of the participants (n = 27, 32.93%) belong to the oldest age group of 51–73 years.

**Table 1:** Characteristics of study population (n = 82).

	Character	Number	Frequency (%)
Gender	Female	66	80.49
	Male	16	19.51
Age	18–30	15	18.29
	31–40	21	25.61
	41–50	19	23.17
	51–73	27	32.93
Occupation	Business	14	17.07
	Farmer	47	57.32
	Student	4	4.88
	Teacher	4	4.88
	Others	13	15.85
Left leg stage	0	12	14.63
	1	7	8.54
	2	31	37.80
	3	32	39.02
	4	0	0
Right leg stage	0	13	15.85
	1	12	14.63
	2	30	36.59
	3	25	30.49
	4	2	2.44

### Microbiome profiles

**Figure 1:**
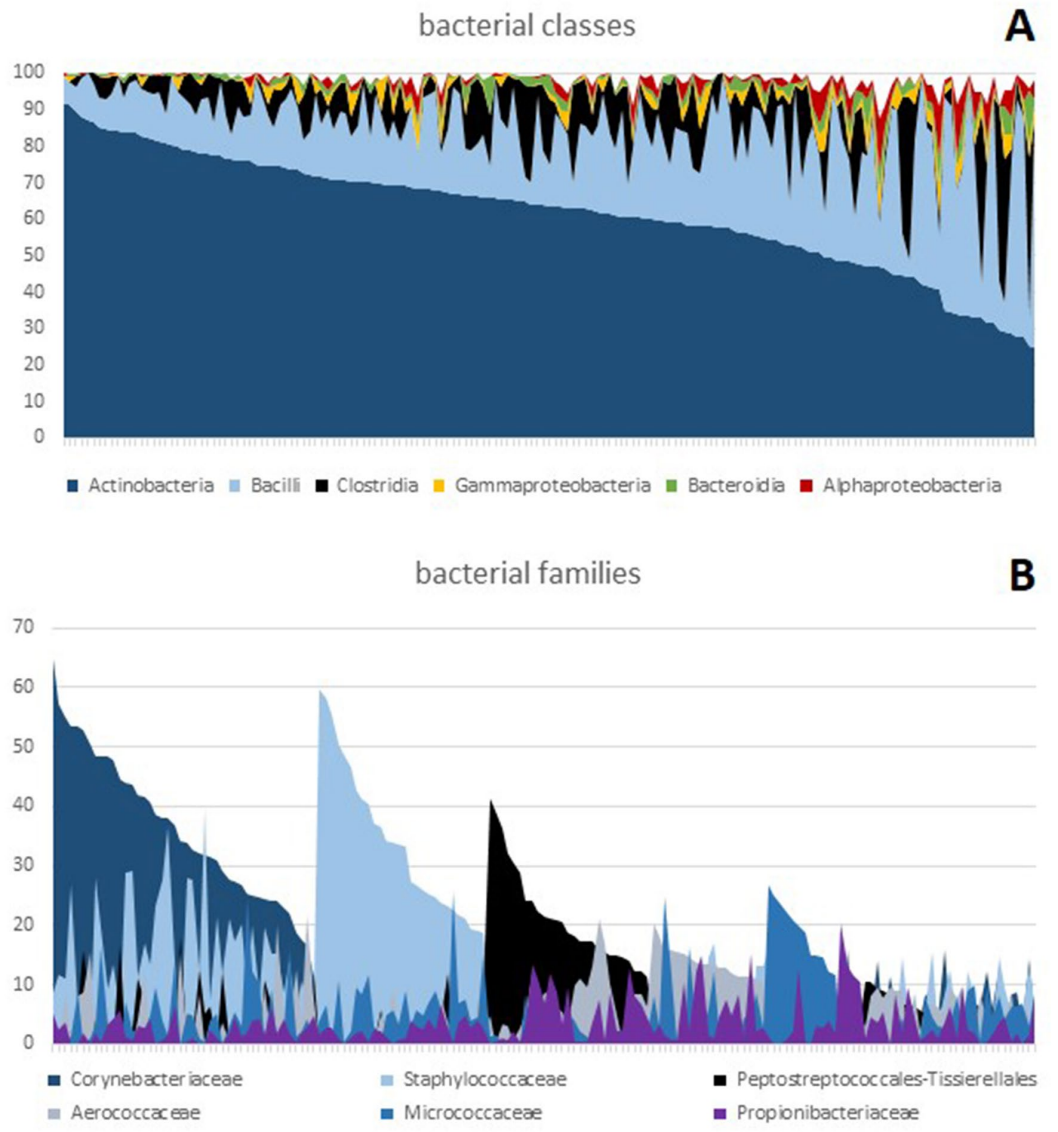
Abundance of bacteria classes and families on the legs of enrolled participants. (**A**) Relative abundance of the six most important bacterial classes. (**B**) Relative abundance of the six most important bacterial families ordered according to the dominant taxa. Each line on the x-axis represents one of the 163 samples.

In total, 164 sequenced skin samples generated a total of 17,791,210 reads with a mean read count of 108,483 per sample. Of these samples, 163 passed the minimum quality filter (> 30,000 reads and > 20,000 merged reads). The 91 most prominent taxa (average prevalence > 0.3%) were considered for statistical analysis (5 phyla, 7 classes, 15 orders, 21 families, 27 genera, 16 species). The most common bacterial class was the Actinobacteria (61.95%). These mainly consisted of *Corynebacteriales* (42.83%) and to a lesser degree of *Staphylococcales* (13.79%) and *Micrococcales* (13.61%). The relative abundance of the six most important bacterial classes and families per sample is depicted in Fig. 1. Based on the predominant bacterial family, the majority of samples could be classified into three primary groups of roughly equal size: those primarily consisting of *Corynebacteriaceae*, those dominated by *Staphylococcaceae*, and those dominated by *Peptostreptococcales-Tissierellales*. A few samples were mildly dominated by *Micrococcaceae* or *Aerococcaceae* and some other samples were without any significant dominant taxa.

### Lymphedema stage and correlation to distinct taxa

**Figure 2:**
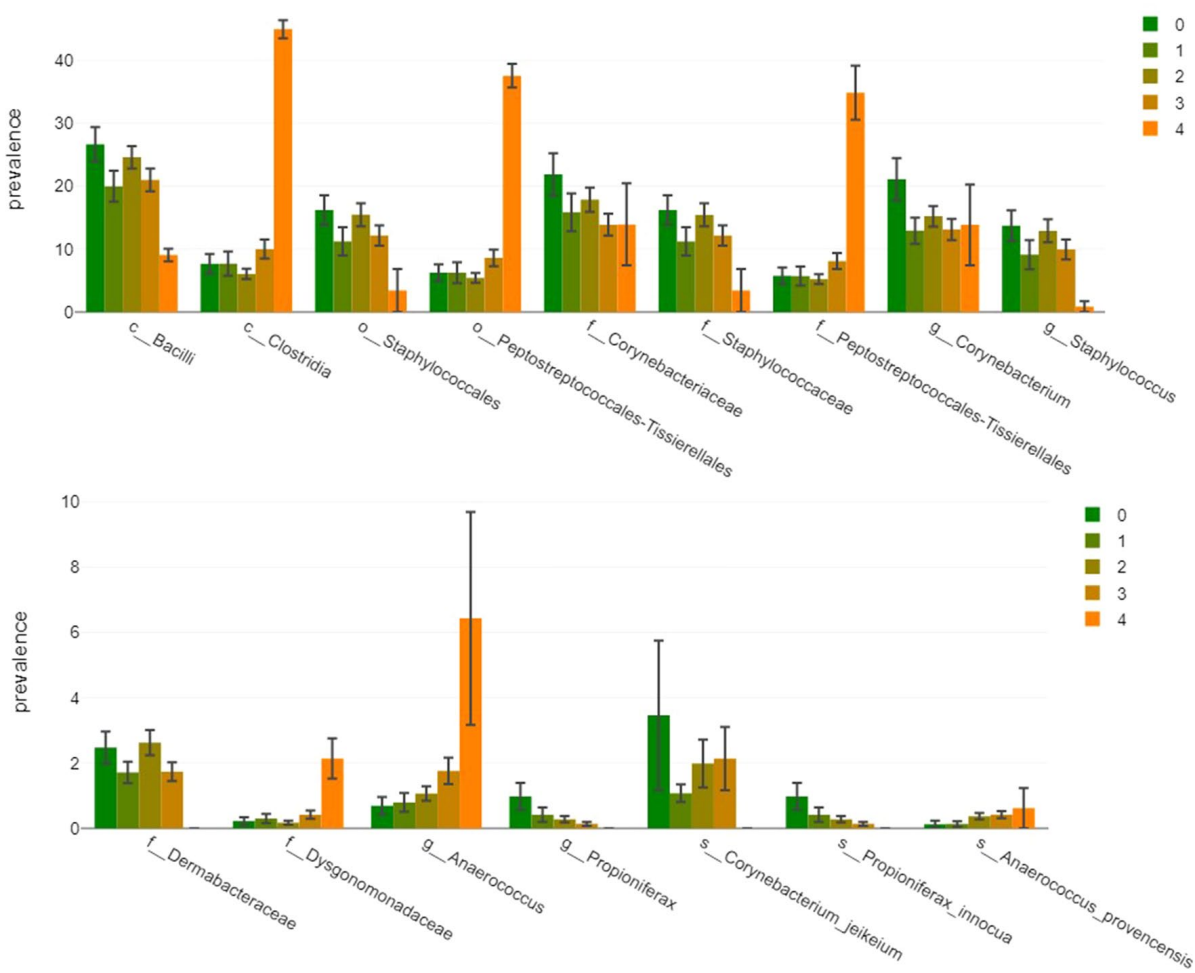
Prevalence of bacteria according to lymphedema stage. Graphs show prevalence of the 16 taxa that were found to be significantly correlated with disease progression by Person correlation, Spearman correlation, or both stratified according to the lymphedema stages (1–4).

**Figure 3:**
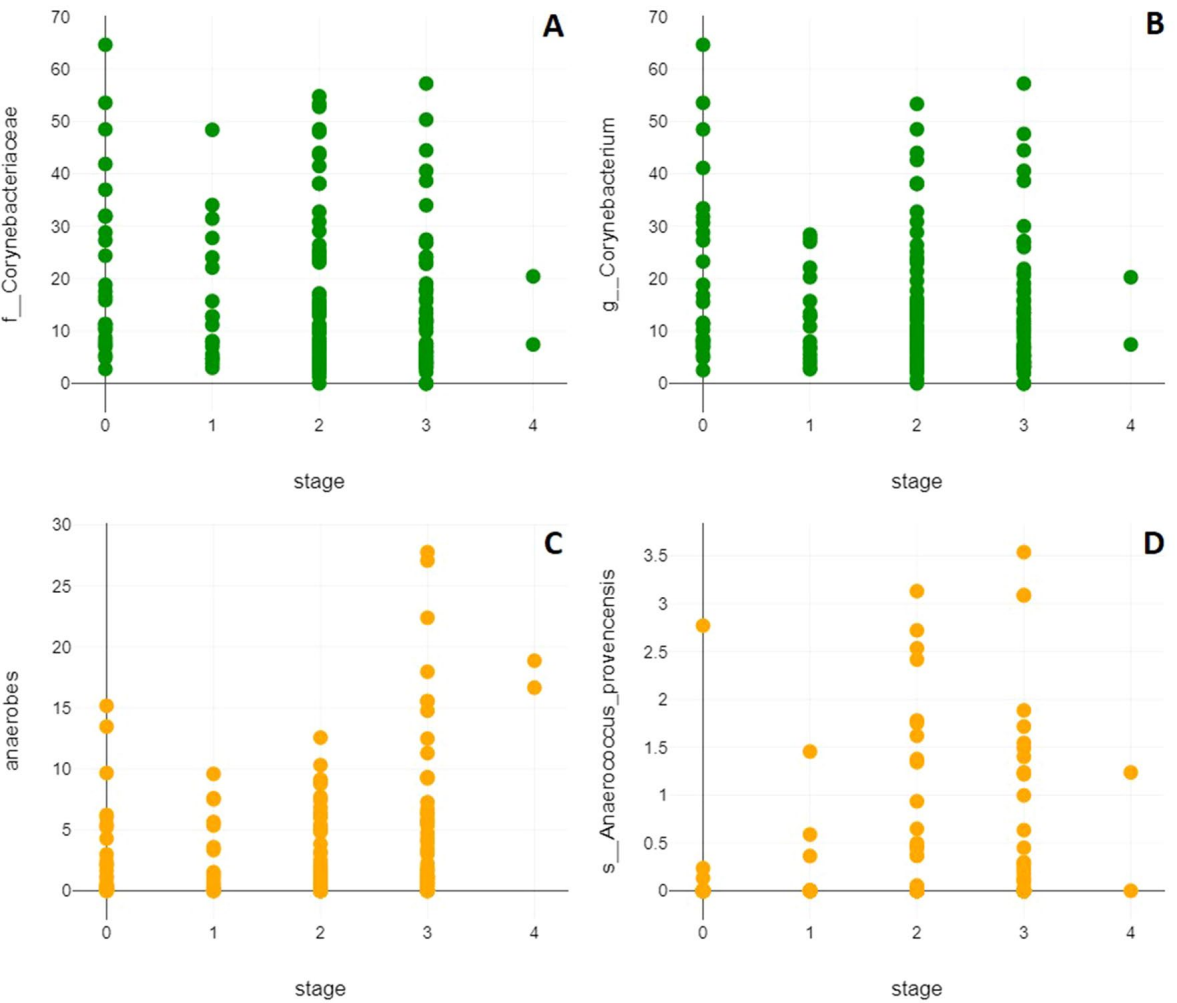
Severity of lymphedema is correlated to distinct bacteria species. (**A**) Increased lymphedema stage is negatively correlated with the family *Corynebacteriaceae* (Pearson: r(161) = −0.17, p = 0.034; Spearman: r(161) = −0.18, p = 0.024) and (**B**) the genus *Corynebacterium* (Pearson: r(161) = −0.17, p = 0.035; Spearman: r(161) = −0.16, p = 0.037), whereas (**C**) the presence of anaerobic genera *Anaerococcus* and *Peptoniphilus* (Pearson: r(161) = 0.2, p = 0.009; Spearman: r(161) = 0.17, p = 0.034) and specifically (**D**) *Anaerococcus provencensis* is positively correlated with increased lymphedema stage (Pearson: r(161) = 0.16, p = 0.045; Spearman: r(161) = 0.21, p = 0.008).

To correlate disease progression with higher or lower abundance of distinct taxa we used both Pearson and Spearman correlation coefficients. This approach accounted for the unique nature of disease stages as both metric and ordinal variables, detected non-linear associations, and ensured reliable results. As depicted in Fig. 2, the results of the Pearson correlations showed that there was a significant positive association between disease stage and the class *Clostridia* (r(161) = 0.17, p = 0.031), the order *Peptostreptococcales-Tissierellales* (r(161) = 0.18, p = 0.021), the family *Peptostreptococcales-Tissierellales* (r(161) = 0.18, p = 0.02), the genus *Anaerococcus* (r(161) = 0.22, p = 0.006), genera other than the 27 evaluated ones (r(161) = 0.16, p = 0.046), and the species *Anaerococcus provencensis* (r(161) = 0.16, p = 0.045). There was a significant negative correlation between disease stage and the family *Corynebacteriaceae* (r(161) = −0.17, p = 0.034), the genera *Corynebacterium* (r(161) = −0.17, p = 0.035) and *Propioniferax* (r(161) = -0.26, p = 0.001), as well as the species *Propioniferax innocua* (r(161) = −0.26, p = 0.001). The results of the Spearman correlations showed that there was a significant positive association between disease stage and the family *Dysgonomonadaceae* (r(161) = 0.16, p = 0.039) and *Anaerococcus provencensis* (r(161) = 0.21, p = 0.008), while there was a significant negative association between disease stage and the class *Bacilli* (r(161) = −0.18, p = 0.02), the order *Staphylococcales* (r(161) = −0.16, p = 0.038), the families *Corynebacteriaceae* (r(161) = −0.18, p = 0.024), *Staphylococcaceae* (r(161) = −0.16, p = 0.037), and *Dermabacteraceae* (r(161) = −0.17, p = 0.028), the genera *Corynebacterium* (r(161) = −0.16, p = 0.037) and *Staphylococcus* (r(161) = −0.19, p = 0.017), as well as the species *Corynebacterium jeikeium* (r(161) = −0.18, p = 0.02). Hence, both correlation coefficients revealed that the family of *Corynebacteriaceae*, the genus *Corynebacterium* and the species *Ananerococcus provencensis* were correlated with disease progression. Moreover, there was a significant positive association between increasing lymphedema stage and the most important anaerobic genera *Peptoniphilus* and *Anaerococcus* taken together in both the Pearson (r(161) = 0.2, p = 0.009) and Spearman correlation (r(161) = 0.17, p = 0.034) (Fig. 3). No other taxa correlations reached the significance threshold and no significant correlations were observed in terms of richness, Shannon- or Simpson-diversity index or evenness.

### Clinical picture and correlation to distinct taxa

A two-tailed t-test for independent samples showed that individuals that suffered from ADLA attacks in the year previous to sample collection had a significantly richer microbiome (taxa: t(118.84) = −2.94, p = 0.004; OTU: t(124.78) =  −2.68, p = 0.008) and a higher Shannon- (taxa: t(104.37) = −2.38, p = 0.019; OTU: t(114.63) = 2.19, p = 0.03) and Fisher-alpha-diversity (taxa: t(119.47) = -3.01, p = 0.003; OTU: t(126.23) = −2.74, p = 0.007). These participants were less prominently colonized by the order *Corynebacteriales* (t(99.62) = 2.41, p = 0.018) and more prominently colonized by the phylum *Deinococcota* (t(138.54) = −2.6, p = 0.01), the classes *Alphaproteobacteria* (t(129.38) = −2.4, p = 0.018) and *Deinococci* (t(138.54) = −2.6, p = 0.01), the order *Micrococcales* (t(127.72) = −2.9, p = 0.004), *Rhizobiales* (t(141) = −2.49, p = 0.014), *Deinococcales* (t(138.55) = −2.6, p = 0.01), and *Sphingomonadales* (t(130.88) = −2.7, p = 0.008), the families *Intrasporangiaceae* (t(134.9) = −5.23, p < 0.001), *Nocardiaceae* (t(117.01) = −4.67, p < 0.001), *Dermacoccaceae* (t(139.6) = −2.96, p = 0.004), *Deinococcaceae* (t(138.9) = −2.57, p = 0.011), and *Sphingomonadaceae* (t(130.88) = −2.7, p = 0.008), the genera *Micrococcus* (t(137.25) = −3.58, p < 0.001), *Kocuria* (t(138.53) = −3.01, p = 0.003), *Janibacter* (t(138.51) = −3.81, p < 0.001), *Nocardioides* (t(114.48) = −4.75, p < 0.001), *Deinococcus* (t(138.9) = −2.57, p = 0.011), *Tessaracoccus* (t(140.95) = −2.55, p = 0.012), *Ornithinimicrobium* (t(129.25) = −5.56, p < 0.001), and *Microbacterium* (t(125.72) = −3.33, p = 0.001), the species *Staphylococcus hominis* (t(112.53) = −2.96, p = 0.004), and phyla (t(124.49) = −2.42, p = 0.017), classes (t(118.55) = −2.52, p = 0.013), and orders (t(112.03) = −2.75, p = 0.007) other than those considered for statistical analysis.

Pearson correlation analysis revealed the number of the ADLA attacks in the year previous to sampling to be negatively correlated with *Lactobacillales* (r(135) = 0.26, p = 0.002) and *Microbacteriaceae* (r(141) = −0.17, p = 0.038) and positively with *Aerococcaceae* (r(135) = 0.24, p = 0.005), *Micrococcaceae* (r(135) = 0.17, p = 0.042), and *Dermabacter hominis* (r(135) = 0.2, p = 0.018). The duration of the last ADLA attacks positively correlated with *Intrasporingiaceae* (r(135) = 0.22, p = 0.011), *Dermacoccaceae* (r(135) = 0.2, p = 0.022), *Brachybacterium* (r(135) = 0.24, p = 0.004), *Kocuria* (r(135) = 0.18, p = 0.036), *Janibacter* (r(135) = 0.24, p = 0.004), *Ornithinimicrobium* (r(135) = 0.24, p = 0.004) and species (r(135) = 0.25, p = 0.003) other than those considered for statistical analysis and negatively correlated with *Corynebacteriaceae* (r(135) = −0.24, p = 0.005), *Corynebacterium* (r(135) = −0.19, p = 0.023), and *Dermabacter* (r(135) = −0.23, p = 0.007).

Skin peeling was associated with an increased richness (taxa: t(87.81) = 3.57, p = 0.001; OTU: t(83.11) = -3.22, p = 0.002) and higher Shannon- (OTU: t(141) = 2.54, p = 0.012) and Fisher-alpha-diversity (taxa: t(87.75) = 3.53, p = 0.001; OTU: t(82.82) = 3.21, p = 0.002). It was also associated more prominently with *Bacilli* (t(141) = −2.83, p = 0.005), *Staphylococcales* (t(141) = −3.64, p < 0.001), *Enterobacterales* (t(141) = −2.06, p = 0.041), *Rhizobiales* (t(66.29) = −2.46, p = 0.016), *Sphingomonadales* (t(94.72) = −2.24, p = 0.028), *Staphylococcaceae* (t(141) = −3.64, p < 0.001), *Sphingomonadaceae* (t(94.72) = −2.24, p = 0.028), *Staphylococcus* (t(97.62) = −2.57, p = 0.012), *Macrococcus* (t(69.85) = −2.3, p = 0.025), *Staphylococcus pettenkoferi* (t(58.26) = −2.22, p = 0.03), and *Brachybacterium conglomeratum* (t(89.84) = −2.82, p = 0.006), and less prominently with *Clostridia* (t(140.99) = 2.64, p = 0.009), order and family *Peptostreptococcales-Tissierellales* (t(140.74) = 2.51, p = 0.013), *Bacteroidales* (t(108.97) = 2.66, p = 0.009), *Dysgonomonadaceae* (t(111.35) = 3.45, p = 0.001), *Peptoniphilus* (t(140.6) = 2.1, p = 0.038), *Gallicola* (t(139.43) = 2.79, p = 0.006), *Tessaracoccus* (t(120.12) = 3.11, p = 0.002), and *Ignavigranum ruoffiae* (t(139.24) = 3.04, p = 0.003).

Finally, fever was associated with a higher richness (taxa: t(42.9) = 4.79, p < 0.001; OTU: *t*(44.15) = 4.88, *p* =  < 0.001), a higher Shannon- (taxa: t(34.45) = 4.51, p < 0.001; OTU: *t*(47.93) = 6.37, *p* < 0.001), Simpson- (taxa: t(17.42) = 2.95, p = 0.009; OTU: *t*(48) = 4.91, *p* < 0.001), and Fisher-alpha-diversity (taxa: t(41.97) = 5.04, p < 0.001; OTU: t(41.14) = 5.45, p < 0.001), and a higher evenness (OTU: t(48) = 2.08, p = 0.043). Furthermore, fever was associated more prominently with *Pseudomonadota* (t(46.85) = −4.13, p < 0.001), *Bacteroidota* (t(47.88) = −2.79, p = 0.007), *Gammaproteobacteria* (t(47.96) = −3.89, p < 0.001), *Bacteroidia* (t(47.88) = −2.79, p = 0.007), *Alphaproteobacteria* (t(46.76) = −3.39, p = 0.001), *Rhodobacterales* (t(47.72) = −2.59, p = 0.013), *Pseudomonadales* (t(47.45) = −3.21, p = 0.002), *Rhizobiales* (t(44.48) = −2.69, p = 0.01), *Sphingomonadales* (t(46.7) = −3.55, p = 0.001), *Flavobacteriales* (t(45.99) = −2.87, p = 0.006), *Intrasporingiaceae* (t(43.24) = −3.51, p = 0.001), *Rhodobacteracea* (t(47.72) = −2.59, p = 0.013), *Pseudomonadaceae* (t(47.34) = −2.74, p = 0.009), *Sphingomonadaceae* (t(46.7) = −3.55, p = 0.001), *Micrococcus* (t(45.88) = −2.45, p = 0.018), *Janibacter* (t(47.93) = −2.76, p = 0.008), *Pseudomonas* (t(47.23) = –2.7, p = 0.01), *Ornithinimicrobium* (t(36.78) = −2.13, p = 0.04), *Microbacterium* (t(46.9) = −2.49, p = 0.016), *Corynebacterium resistens* (t(35) = −2.3, p = 0.027) and classes (t(42.32) = −2.41, p = 0.021) other than those considered for statistical analysis.

A summary of all correlations between leg skin microbiome and clinical symptoms of podoconiosis individuals is shown in Table 2.

**Table 2:** Correlations of clinical symptoms and leg skin microbiome of podoconiosis individuals.

Characteristics	Positively associated	Negatively associated
Presence of ADLA attack 1 year before sampling	Richness and diversity of leg skin microbiome Phyla*: Deinococcota* Classes*: Alphaproteobacteria, Deinococci* Orders*: Micrococcales, Rhizobiales, Deinococcales, Sphingomonadales* Families*: Intrasporangiaceae, Nocardiaceae, Dermacoccaceae, Deinococcaceae, Sphingomonadaceae* Genera*: Micrococcus, Kocuria, Janibacter, Nocardioides, Deinococcus, Tessaracoccus, Ornithinimicrobium, Microbacterium* Species*: Staphylococcus hominis*	Orders*: Corynebacteriales*
Number of last ADLA attacks	Families*: Intrasporangiaceae, Micrococcaceae* Species*: Dermabacter hominis*	Orders*:* *Lactobacillales* Families*: Microbacteriaceae*
Duration of last ADLA attack	Families*: Intrasporangiaceae, Dermacoccaceae* Genera*: Brachybacterium, Kocuria, Janibacter, Ornithinimicrobium*	Families*: Corynebacteriaceae* Genera*: Corynebacterium, Dermabacter*
Skin peeling	Richness and diversity of leg skin microbiome Classes*: Bacilli* Orders*: Staphylococcales, Enterobacterales, Rhizobiales, Sphingomonadales* Families*: Staphylococcaceae, Sphingomonadaceae* Genera*: Staphylococcus, Macrococcus* Species*: Staphylococcus pettenkoferi, Brachybacterium conglomeratum*	Classes*: Clostridia* Orders*: Peptostreptococcales-Tissierellales, Bacteroidales* Families*: Peptostreptococcales-Tissierellales, Dysgonomonadaceae* Genera*: Peptoniphilus, Gallicola, Tessaracoccus* Species*: Ignavigranum ruoffiae*
Fever	Richness, diversity and evenness of leg skin microbiome Phyla*: Pseudomonadota, Bacteroidota,* Classes*: Gammaproteobacteria, Bacteroidia, Alphaproteobacteria,* Orders*: Rhodobacterales, Pseudomonadales, Rhizobiales, Sphingomonadales, Flavobacteriales* Families*: Intrasporangiaceae, Rhodobacteraceae, Pseudomonaceae, Sphingomonadaceae* Genera*: Micrococcus, Janibacter, Pseudomonas, Ornithinomicrobium, Microbacterium* Species*: Corynebacterium resistens*	

## Discussion

The microbiome of the skin varies greatly depending on the anatomical location. The toe web space has been described as being represented mainly by *Corynebacteriaceae*, and to a slightly lesser degree, by *Micrococcaceae* and *Staphylococcaceae*^22,23^. These three bacterial families represent three out of the five most prominently detected ones in our study. *Peptostreptococcales-Tissierellales* are not frequently found in human skin microbiome analyses, but represent the third most common family in our study. One cannot rule out the possibility that Cameroonians may simply be underrepresented in skin microbiome studies to date. The only study we found that specifically looked at the skin microbiome of Cameroonians included 27 healthy Cameroonians and compared this to that of healthy Japanese^24^. The taxa that were correlated with progressive disease stages in our study were either absent or present at very low levels in the skin microbiomes of these healthy Cameroonians. The high prevalence of *Peptostreptococcales-Tissierellales* may thus be more closely related to the disease of the patient group studied than to their geography. Anaerobic bacteria other than *Cutibacterium acnes* and some anaerobic *Staphylococci*, are rarely detected in this context and mostly associated with disease or chronic wounds^22,23,25,26^. *Staphylococci* have been already isolated from wounds of patients with lower limb lymphedema from Ethiopia^27^. Future studies need to be performed to analyse if the microbiome differs between wounds and skin of the affected legs. Nevertheless, available literature is far from exhaustive. The fact that our study mainly included individuals affected by podoconiosis at different disease stages could explain this finding. Our study revealed a significant correlation between disease progression and the rising prevalence of anaerobic gram-positive cocci, such as *Anaerococcus provencensis*. This bacterium was first isolated from a cerebral abscess sample of a patient from Marseille^28^, while scarcely anything else is known about this pathogen. As with all 16s amplicon studies, even significant taxa should be interpreted with caution despite the suitably large sample size. Nevertheless, we do see a trend in regards to disease symptoms which were all associated with a higher microbial richness and diversity. In particular long and frequent ADLA attacks and fever were associated with smaller proportions of microbes usually expected on intact skin and higher prevalence of bacteria that possibly do not belong on the skin.

Previous studies found *Staphylococcus*, *Enterococcus*, *Micrococcus* and *Bacillus* spp. in tissue and lymph fluid as well as lymph nodes from lymphatic filariasis patients and suggested that these bacteria may be responsible for progression of lymphedema and ADLA attacks^20,21^. However, future investigations need to be performed to narrow down distinct bacterial species that are important for the progression of the disease by collecting lymph fluid or blood of individuals with different lymphedema stages before and during ADLA attacks. The investigation of distinct bacterial patterns might also reveal potential biomarkers for the discrimination of podoconiosis and lymphatic filariasis from other infectious and non-infectious forms of lymphedema. Nevertheless, this study has some limitations such as the small number of individuals with high lymphedema stages (especially leg stage 4) and thus, future studies should obtain samples from stage 4 and 5 to proof the positive correlation of anaerobic bacteria and severity of lymphedema. In addition, the majority of enrolled participants were women (80%). Indeed, previous publications already showed that women are more affected than men^4,29–32^, since they often have less income than men and thus cannot afford proper shoes and moreover, spent longer periods of time in the fields and therefore are more exposed to irritant soil^31,33^. Further studies should focus on sampling additional skin sites and most importantly skin folds for detailed discrimination of skin bacteria and should include more male participants to decipher if gender influences the skin microbiome of podoconiosis legs.

In summary, we conclude that the microbiome of the skin from the ankle to the interdigital spaces of podoconiosis individuals with steady deterioration is increasingly characterized by the presence of anaerobic bacteria, which are not described to be part of the healthy skin flora. Whether and to what extent these bacteria are responsible for the fever episodes or the progression of the disease is urgently to be clarified, as tailored treatment options would most likely not be challenging to implement.

## Methods

### Ethics approval and consent to participate

The ethical clearance was sought and obtained from the Faculty of Health Sciences Institutional Review Board of University of Buea (No: 2021/1458-05/UB/SG/IRB/FHS) and the Ethics Ctwoommittee at the University Hospital Bonn, Germany (Lfd Nr. 359/17). The administrative clearance was obtained from North West Regional Delegation of Public Health (No: 2021/203/ATT/NWR/RDPH/BRIGAD). All experiments and methods were performed in accordance with the relevant guidelines and regulations.

### Study area and design

This cross-sectional study was conducted at the Bamenda Clinical Trial Centre (BCTC) in NRW of Cameroon, which is characterised by a mean altitude of 1403 m above sea level, mean annual rainfall of 2500 mm and presence of fertile clay soils used to grow rice, maize, beans and other cash crops by farmers^34^. This region has been shown to have the highest prevalence of podoconiosis among 10 regions of Cameroon^7,35^.

### Study population

Podoconiosis individuals and contacts without lymphedema living in the same communities visiting the BCTC were recruited in this study. Eligible individuals had to be at least 18 years old, to have lived in the NWR for at least 2 years, to not be pregnant and to have no evidence of severe or systematic co-morbidities. Leg stages of the individuals affected by podoconiosis were classified according to the de novo clinical staging system^14^. In addition, we enrolled 8 lymphedema-free individuals from the same communities and obtained 9 individuals with one healthy leg. Thus, in total we obtained 25 control samples (stage 0). Information about the study was provided and eligible individuals willing to participate in the study signed or provided a thumb print on informed consent forms. Then, a questionnaire-based survey was applied to collect socio-demographic data like gender, age, occupation, duration in the community, and educational level from each participant and medical history especially information about ADLA attacks (number and duration of attacks within the last year before sample collection and occurrence of skin peeling and fever during ADLA attacks), which was then confirmed by medical records, if available.

### Collection of skin swabs

Samples were collected from both legs of each participant by swabbing the skin areas between toes, skin folds and around the anklebone under strict aseptic techniques to avoid contamination using pre-labelled sterile flocked swabs (Copan, Brescia, Italy). In total, we obtained two swabs per participant (one swab per leg). The swabs were immediately transferred into 1 ml eNAT medium tubes and stored at −20 °C. For consecutive analysis samples were shipped to the Institute for Medical Microbiology, Immunology and Parasitology (IMMIP) at the University Hospital Bonn (UKB), Bonn, Germany.

### Skin swab DNA extraction and sequencing

Skin swab samples were homogenized using the bead beating precellys evolution homogenizer (Bertin Technologies SAS Bretonneux, France). Highly purified DNA was then extracted using the column-based PureLink Microbiome DNA Purification Kit (Thermo Fisher Scientific, Waltham, MA, USA) according to the manufacturer’s instructions. At the end of the extraction process the DNA was eluted to 100 µL volume and qualitatively and quantitatively evaluated using the NanoDrop OneC (Thermo Fisher Scientific). Then, 16S rRNA gene sequencing libraries were constructed from each sample using the Quick-16S NGS Library Prep Kit (Zymo Research Europe GmbH, Freiburg, Germany) with its included optimized V1–V2 primer pairs. Each run included 94 samples, the positive control included in the kit, and a negative control. For quantitative PCR, quality control, and normalization purposes the Bio-Rad CFX96 Real-Time PCR Detection System (Bio-Rad Laboratories, Inc., Hercules, California, USA) was used. After pooling, the DNA was quantified with the QuantiFluor dsDNA System on the Quantus Fluorometer (Promega GmbH, Walldorf, Germany) and diluted strictly according to the Illumina protocol for MiSeq sample preparation. For the final library a loading concentration of 10pm was chosen and a 10% Illumina v3 PhiX spike-in control added before running it on the Illumina MiSeq platform with a 500cycle v2 Illumina MiSeq Reagent Kit. All reagents and equipment for sequencing of samples were obtained from Illumina, San Diego, CA, USA.

### Bioinformatic analysis

The bioinformatic analysis included three main parts, starting with the preprocessing of raw paired end reads. Following the preprocessing, the sequences were assigned to taxonomies. Finally, a statistical and graphical evaluation was performed on the resulting taxa. QIIME2^36^ was used for both preprocessing and classification of the data. With the plugin tool DADA2^37^ forward and reverse reads were trimmed from the 3’ end at position 249, while shorter reads, as well as low-quality reads, got discarded. DADA2 was also used to perform error-correction, merging of forward and reverse reads if there was an overlap of at least 12 base pairs and chimera removal.

The processed sequences were clustered into OTUs (operational taxonomic units) of 100% sequence identity and assigned to taxa, using a classifier trained on full-length sequences of SILVA^38^. The trained classifier was provided by QIIME2 using scikit-learn 0.24.1 and the plugin tool q2-feature-classifier^39,40^. Based on the quantified OTUs and taxa, different diversity indices were calculated using Python and the skbio.diversity library. The Richness, Shannon-, Simpson- and Fisher-index as a measurement for alpha diversity and the Bray–Curtis- and Jaccard-index as a measurement for beta diversity. The Shannon index quantifies the level of entropy in predicting the species identity of a randomly selected individual from a community, whereas the Simpson index assesses the likelihood that two randomly selected individuals belong to the same species. Fisher's alpha is a measure of species richness that adjusts for variations in sample size. The relative abundance of the taxa and the measurements of alpha diversity were tested for statistically significant differences between groups of samples. This analysis was performed in Python using the Mann–Whitney-U test from the statsmodels library^41^. Additionally, taxa frequency comparison and correlation analysis were performed using Datatab version 1.12.1. with the Pearson correlation coefficient used for continuous and normally distributed variables and Spearman correlation coefficient used for ordinal or non-normally distributed variables.

## Data Availability

The datasets generated during and/or analysed during the current study are not publicly available due to ethical restrictions since they contain information that could compromise the privacy of research participants but are available from the corresponding author on reasonable request.

## References

[1] Price E (1990). Podoconiosis: Non-Filarial Elephantiasis.

[2] Davey G (2012). Launch of the international podoconiosis initiative. Lancet.

[3] Semrau M (2019). Depressive symptoms amongst people with podoconiosis and lower limb lymphoedema of other cause in Cameroon: A cross-sectional study. Trop. Med. Infect. Dis..

[4] Wanji S (2008). Elephantiasis of non-filarial origin (podoconiosis) in the highlands of North-Western Cameroon. Ann. Trop. Med. Parasit..

[5] Wanji S (2021). Podoconiosis—From known to unknown: Obstacles to tackle. Acta Trop..

[6] Deribe K (2017). The global atlas of podoconiosis. Lancet Glob. Health..

[7] Deribe K (2018). Predicted distribution and burden of podoconiosis in Cameroon. BMJ Glob. Health..

[8] Deribe K (2019). Mapping the global distribution of podoconiosis: Applying an evidence consensus approach. PLoS Negl. Trop. Dis..

[9] Price EW (1976). The association of endemic elephantiasis of the lower legs in East Africa with soil derived from volcanic rocks. Trans. R. Soc. Trop. Med. Hyg..

[10] Deribe K (2015). Epidemiology and individual, household and geographical risk factors of podoconiosis in Ethiopia: Results from the first nationwide mapping. Am. J. Trop. Med. Hyg..

[11] Deribe K, Cano J, Trueba ML, Newport MJ, Davey G (2018). Global epidemiology of podoconiosis: A systematic review. PLoS Negl. Trop. Dis..

[12] Wanji S (2018). Study of lymphoedema of non-filarial origin in the northwest region of Cameroon: Spatial distribution, profiling of cases and socio-economic aspects of podoconiosis. Int. Health..

[13] Deribe K (2020). Developing and validating a clinical algorithm for the diagnosis of podoconiosis. Trans. R. Soc. Trop. Med. Hyg..

[14] Tekola F, Ayele Z, Mariam DH, Fuller C, Davey G (2008). Development and testing of a de novo clinical staging system for podoconiosis (endemic non-filarial elephantiasis). Trop. Med. Int. Health..

[15] Fuller LC (2005). Podoconiosis: Endemic nonfilarial elephantiasis. Curr. Opin. Infect. Dis..

[16] Wendemagegn E, Tirumalae R, Böer-Auer A (2015). Histopathological and immunohistochemical features of nodular podoconiosis. J. Cutan. Pathol..

[17] Ferguson JS, Yeshanehe WE, Matts PJ, Davey G, Mortimer PS, Fuller LC (2013). Assessment of skin barrier function in podoconiosis: Measurement of stratum corneum hydration and transepidermal water loss. Br. J. Dermatol..

[18] Brooks J, Ersser SJ, Cowdell F, Gardiner E, Mengistu A, Matts PJ (2017). A randomized controlled trial to evaluate the effect of a new skincare regimen on skin barrier function in those with podoconiosis in Ethiopia. Br. J. Dermatol..

[19] Phillips, C., Samuel, A., Tiruneh, G., Deribe, K. &, Davey, G. The impact of acute adenolymphangitis in podoconiosis on caregivers: a case study in Wayu Tuka Woreda, Oromia, Western Ethiopia. “If she was healthy, I would be free. *PLoS Negl. Trop. Dis.* **13**, e0007487 (2019). 10.1371/journal.pntd.0007487 PMC6638979 31283763

[20] Olszewski WL (1999). Bacteriological studies of blood, tissue fluid, lymph and lymph nodes in patients with acute dermatolymphangioadenitis (DLA) in course of 'filarial' lymphedema. Acta Trop..

[21] Olszewski WL (1997). Bacteriologic studies of skin, tissue fluid, lymph, and lymph nodes in patients with filarial lymphedema. Am. J. Trop. Med. Hyg..

[22] Grice EA, Segre JA (2011). The skin microbiome. Nat. Rev. Microbiol..

[23] Byrd AL, Belkaid Y, Segre JA (2018). The human skin microbiome. Nat. Rev. Microbiol..

[24] Naik HB, Piguet V (2020). Standardizing hidradenitis suppurativa skin microbiome research: The methods matter. J. Invest. Dermatol..

[25] Ogai K (2022). Skin microbiome profile of healthy Cameroonians and Japanese. Sci. Rep..

[26] Choi Y (2019). Co-occurrence of anaerobes in human chronic wounds. Microb. Ecol..

[27] Nigussie D (2020). Antimicrobial susceptibility of bacteria isolated from the infected wounds of patients with lymphoedema in East Wollega, Ethiopia. Trans. R Soc. Trop. Med. Hyg..

[28] Pagnier I (2014). Non-contiguous finished genome sequence and description of Anaerococcus provenciensis sp. nov.. Stand Genomic Sci..

[29] Davey G (2007). Podoconiosis: Non-infectious geochemical elephantiasis. Trans. R Soc. Trop. Med. Hyg..

[30] Alemu G (2011). Burden of podoconiosis in poor rural communities in Gulliso woreda, west Ethiopia. PLoS Negl. Trop. Dis..

[31] Molla YB (2013). Individual correlates of podoconiosis in areas of varying endemicity: A case–control study. PLoS Negl. Trop. Dis..

[32] Tekola F (2006). Economic costs of endemic non-filarial elephantiasis in Wolaita Zone, Ethiopia. Trop. Med. Int. Health.

[33] Kihembo C (2017). Risk factors for podoconiosis: Kamwenge District, Western Uganda, September 2015. Am. J. Trop. Med. Hyg..

[34] Wanji S (2016). Detecting and staging podoconiosis cases in North West Cameroon: Positive predictive value of clinical screening of patients by community health workers and researchers. BMC Public Health.

[35] Deribe K (2018). Mapping the geographical distribution of podoconiosis in Cameroon using parasitological, serological, and clinical evidence to exclude other causes of lymphedema. PLoS Negl. Trop. Dis..

[36] Bolyen E (2019). Reproducible, interactive, scalable, and extensible microbiome data science using QIIME 2. Nat. Biotech..

[37] Callahan BJ, McMurdie PJ, Rosen MJ, Han AW, Johnson AJ, Holmes SP (2016). DADA2: High-resolution sample inference from Illumina amplicon data. Nat. Methods.

[38] Quast C (2013). The SILVA ribosomal RNA gene database project: Improved data processing and web-based tools. Nucl. Acids Res..

[39] Robeson MS (2021). RESCRIPt: Reproducible sequence taxonomy reference database management. PLoS Comput. Biol..

[40] Bokulich NA (2018). Optimizing taxonomic classification of marker-gene amplicon sequences with QIIME 2's q2-feature-classifier plugin. Microbiome..

[41] Seabold, S. & Perktold, J. Statsmodels: Econometric and statistical modeling with python. in *9th Python in Science Conference* (2010).

